# Laboratory Surveillance of Influenza-Like Illness in Seven Teaching Hospitals, South Korea: 2011–2012 Season

**DOI:** 10.1371/journal.pone.0064295

**Published:** 2013-05-22

**Authors:** Ji Yun Noh, Joon Young Song, Hee Jin Cheong, Won Suk Choi, Jacob Lee, Jin-Soo Lee, Seong-Heon Wie, Hye Won Jeong, Young Keun Kim, Sung Hyuk Choi, Seung Baik Han, Byung-Hak So, Hyun Kim, Woo Joo Kim

**Affiliations:** 1 Department of Microbiology, Korea University College of Medicine, Seoul, Korea; 2 Division of Infectious Diseases, Department of Internal Medicine, Korea University Guro Hospital, Korea University College of Medicine, Seoul, Korea; 3 Asia Pacific Influenza Institute, Korea University College of Medicine, Seoul, Korea; 4 Division of Infectious Diseases, Department of Internal Medicine, Korea University Ansan Hospital, Korea University College of Medicine, Ansan, Gyeonggi province, Korea; 5 Division of Infectious Diseases, Department of Internal Medicine, Kangnam Sacred Heart Hospital, Hallym University School of Medicine, Seoul, Korea; 6 Division of Infectious Diseases, Department of Internal Medicine, Inha University College of Medicine, Incheon, Korea; 7 Division of Infectious Diseases, Department of Internal Medicine, The Catholic University of Korea, School of Medicine, St. Vincent’s Hospital, Suwon, Gyeonggi province, Korea; 8 Division of Infectious Diseases, Department of Internal Medicine, College of Medicine, Chungbuk National University, Cheongju, Chungcheongbuk province, Korea; 9 Department of Infectious Disease, Yonsei University Wonju College of Medicine, Wonju, Gangwon province, Korea; 10 Department of Emergency Medicine, Korea University Guro Hospital, Korea University College of Medicine, Seoul, Korea; 11 Department of Emergency Medicine, College of Medicine, Inha University, Incheon, Korea; 12 Department of Emergency Medicine, The Catholic University of Korea, School of Medicine, St. Vincent’s Hospital, Suwon, Gyeonggi province, Korea; 13 Department of Emergency Medicine, Yonsei University Wonju College of Medicine, Wonju, Gangwon province, Korea; 14 Transgovernmental Enterprise for Pandemic Influenza in Korea, Seoul, Korea; University of Hong Kong, Hong Kong

## Abstract

**Background:**

A well-constructed and properly operating influenza surveillance scheme is essential for public health. This study was conducted to evaluate the distribution of respiratory viruses in patients with influenza-like illness (ILI) through the first teaching hospital-based surveillance scheme for ILI in South Korea.

**Methods:**

Respiratory specimens were obtained from adult patients (≥18 years) who visited the emergency department (ED) with ILI from week 40, 2011 to week 22, 2012. Multiplex PCR was performed to detect respiratory viruses: influenza virus, adenovirus, coronavirus, respiratory syncytial virus, rhinovirus, human metapneumovirus, parainfluenza virus, bocavirus, and enterovirus.

**Results:**

Among 1,983 patients who visited the ED with ILI, 811 (40.9%) were male. The median age of patients was 43 years. Influenza vaccination rate was 21.7% (430/1,983) during the 2011–2012 season. At least one comorbidity was found in 18% of patients. The positive rate of respiratory viruses was 52.1% (1,033/1,983) and the total number of detected viruses was 1,100. Influenza A virus was the dominant agent (677, 61.5%) in all age groups. The prevalence of human metapneumovirus was higher in patients more than 50 years old, while adenovirus was detected only in younger adults. In 58 (5.6%) cases, two or more respiratory viruses were detected. The co-incidence case was identified more frequently in patients with hematologic malignancy or organ transplantation recipients, however it was not related to clinical outcomes.

**Conclusion:**

This study is valuable as the first extensive laboratory surveillance of the epidemiology of respiratory viruses in ILI patients through a teaching hospital-based influenza surveillance system in South Korea.

## Introduction

Surveillance for influenza is indispensable for the timely monitoring and response to outbreaks or epidemics of influenza [1]. Circulating influenza viruses can be identified through a systemized surveillance program. In addition, the data from influenza surveillance are applicable to evaluating vaccine effectiveness and to selecting vaccine strains [2]. Thus, a well-constructed and properly operating influenza surveillance scheme is essential for public health [3].

The annual epidemics of respiratory viruses including influenza virus are various by countries and region. Large-scaled study on epidemiology of respiratory viruses is important to understand the situation of individual country. Previously, a retrospective analysis of the respiratory viruses was performed in patients with respiratory illnesses in South Korea [4]. Respiratory viral etiology has been investigated in hospitalized neonates and in adults with pneumonia requiring intensive care also [5], [6]. However, there is a lack of information regarding large-scaled multi-center viral surveillance of adult patients with ILI (influenza-like illness) in emergency department (ED).

This study involves the first extensive laboratory surveillance of ILI patients through the first teaching hospital-based influenza surveillance scheme in South Korea during the 2011–2012 influenza season. This study aimed to investigate the distribution and seasonality of respiratory viruses detected in adult patients with ILI who visited EDs at seven teaching hospitals.

## Materials and Methods

### Ethics Statement

The study protocol was approved by the Institutional Review Board (IRB) in each hospital (approval number): Korea University Guro Hospital (KUGH11088, KUGH12007-001), Korea University Ansan Hospital (AS11047), Hallym University Kangnam Sacred Heart Hospital (2011-06-50), The Catholic University St. Vincent’s Hospital (VC11ONME0118), Inha University Hospital (11-1534), Yonsei University Wonju Christian Hospital (CR311025), and Chungbuk National University Hospital (2011-06-044). All patients gave written informed consent. The study was conducted in accordance with the principles and guidelines expressed in the Declaration of Helsinki.

### Study Population

**Figure 1:**
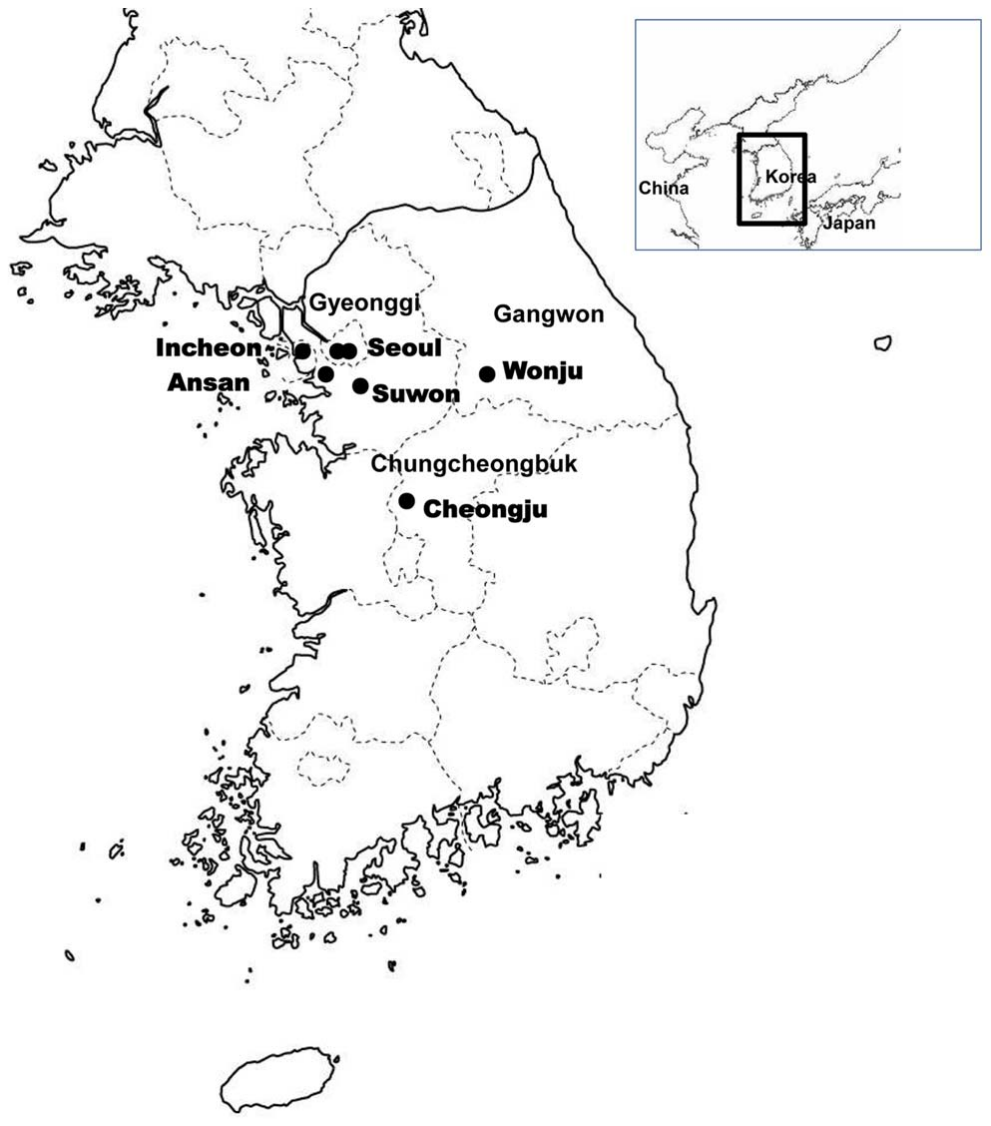
Map of the study sites in South Korea.

Hospital-based Influenza Morbidity and Mortality (HIMM) is the teaching hospital-based clinical and laboratory influenza surveillance scheme in South Korea. A total of seven teaching hospitals participated the scheme in the 2011–2012 influenza season: two hospitals in Seoul, two hospitals in Gyeonggi province (Ansan and Suwon), one hospital in Incheon, one hospital in Gangwon province (Wonju) and one hospital in Chungcheongbuk province (Cheongju). Figure 1 shows a map of the study sites. The study population included adult patients (≥18 years) who visited an ED with ILI from week 40, starting Sep 25, 2011, through week 22, ending Jun 2, 2012. ILI was defined as an acute respiratory illness with measured fever of ≥38°C or afebrile state in the case of antipyretics use in last eight hours and at least one of respiratory symptoms, cough, sore throat, rhinorrhea and/or nasal congestion.

### Specimen Collection

A nasopharyngeal or oropharyngeal flocked swab was obtained from each ILI patient. The flocked swab was placed in viral transport medium (BD, USA) immediately and stored at 4°C while being transferred to the laboratory at Korea University Guro Hospital, Seoul. Samples were then kept at −70°C until use.

### Laboratory Analysis

Total RNA was extracted from the viral transport medium containing the flocked swab using NucliSENS® easyMAG® (BioMérieux, France) as per the manufacturer’s protocol. Reverse transcription was performed using the AccuPower® CycleScript RT PreMix (Bioneer, Korea) and random hexamer was used to synthesize cDNA. All cDNA samples were stored at −20°C until use. Multiplex PCR was performed using Seeplex® RV15 ACE Detection (Seegene, Korea). To detect influenza A virus, influenza B virus, adenovirus, coronavirus OC43, coronavirus 229E/NL63, respiratory syncytial virus (RSV) A, RSV B, rhinovirus, human metapneumovirus, parainfluenza virus type 1, parainfluenza virus type 2, parainfluenza virus type 3, parainfluenza virus type 4, bocavirus, and enterovirus, each sample was tested in three tubes reactions. Briefly, 4 µL 5X RV15 ACE primer mix, 10 µL 2X Multiplex master mix, 3 µL 8-MOP solution, and 3 µL of the sample’s cDNA were mixed in a tube. After denaturation at 94°C for 15 minutes, a PCR reaction was performed using 40 cycles at 94°C for 30 seconds, 60°C for 90 seconds, 72°C for 90 seconds, followed by extension at 72°C for 10 minutes. Among the samples tested, those positive for influenza A virus were selected and were tested to differentiate subtype using Seeplex® Influenza A/B Onestep Typing (Seegene, Korea). Briefly, 7.5 µL 8-MOP solution, 2.5 µL random hexamer, 10 µL 5X Flu A/B One-Step primer mix, 10 µL 5X OneStep RT-PCR buffer, 2 µL OneStep RT-PCR enzyme mix, 8 µL RNase-free water, and 10 µL sample RNA were mixed in the tube. Cycling conditions included a reverse transcription step at 50°C for 30 minutes and a denaturation step at 94°C for 15 minutes. The PCR reaction was performed using 45 cycles at 94°C for 30 seconds, 60°C for 90 seconds, 72°C for 60 seconds, followed by extension at 72°C for 10 minutes. Positive and negative controls were included in each PCR analysis. Electrophoresis of all PCR amplicons was performed on a 1% agarose gel.

### Statistical Analysis

Data are expressed as median with interquartile range (IQR) for continuous variables and count with percent for categorical variables. Fisher’s exact test was used to analyze categorical variables. Statistical analyses were carried out using SPSS 12.0 (SPSS Inc., Chicago, IL, USA) and a *P* value of <0.05 was considered to be statistically significant.

## Results

### Clinical Characteristics of Patients with ILI

From week 40, starting Sep 25, 2011 to week 22, ending Jun 2, 2012, 1,983 patients with ILI visited EDs. The clinical characteristics of the patients are shown in Table 1. The median age of patients was 43 years (IQR 31–63), and 811 patients were male (40.9%). The age distribution was as follows: 461 (23.2%), 18–30 years; 672 (33.9%), 31–49 years; 390 (19.7%), 50–64 years; 460 (23.2%), ≥65 years. At least one chronic underlying disease was found in 356 patients (18.0%). Diabetes mellitus was the most frequent comorbidity (36.8%, 131/356) in patients with ILI. Forty five (12.6%) patients had cardiovascular disease. Among the patients with ILI, there were 32 pregnant patients. Four hundred thirty patients (21.7%) received an influenza vaccination during the 2011–2012 season before the hospital visit. Vaccination rates varied according to age group: 13.2% in 18–30 years (61/461); 13.7% in 31–49 years (92/672); 21.8% in 50–64 years (85/390); 41.7% in ≥65 years (192/460).

**Table 1:** Clinical characteristics of ILI patients from week 40, 2011 through week 22, 2012.

	Total	Hospital A (Seoul)	Hospital B (Seoul)	Hospital C (Suwon)	Hospital D (Ansan)	Hospital E (Wonju)	Hospital F (Incheon)	Hospital G (Cheongju)
Number of patients	1,983	290	77	735	281	44	80	476
Sex (male), n(%)	811 (40.9)	111 (38.3)	30 (39.0)	298 (40.5)	118 (42.0)	23 (52.3)	34 (42.5)	197 (41.4)
Age, median (IQR)	43 (31–63)	35.5 (29–55)	36 (31–55)	46 (32–63)	37 (30–54)	49 (32–67)	41.5 (33–57.5)	53 (33–72)
18–30	461 (23.2)	91 (31.4)	18 (23.4)	154 (21.0)	75 (26.7)	9 (20.5)	15 (18.8)	99 (20.8)
31–49	672 (33.9)	106 (36.6)	35 (45.5)	251 (34.1)	119 (42.3)	13 (29.5)	34 (42.5)	114 (23.9)
50–64	390 (19.7)	56 (19.3)	11 (14.3)	157 (21.4)	49 (17.4)	7 (15.9)	17 (21.3)	93 (19.5)
≥65	460 (23.2)	37 (12.8)	13 (16.9)	173 (23.5)	38 (13.5)	15 (34.1)	14 (17.5)	170 (35.7)
Comorbidities								
unknown	685 (34.5)	203 (70.0)	–	9 (1.2)	–	–	1 (1.3)	472 (99.2)
none	942 (47.5)	49 (16.9)	62 (80.5)	533 (72.5)	216 (76.9)	29 (65.9)	53 (66.3)	–
yes	356 (18.0)	38 (13.1)	15 (19.5)	193 (26.3)	65 (23.1)	15 (34.1)	26 (32.5)	4 (0.8)
DM	131 (36.8)	10 (26.3)	5 (33.3)	83 (43.0)	21 (32.3)	4 (26.7)	5 (19.2)	3 (75.0)
Cardiovascular disease	45 (12.6)	7 (18.4)	3 (20.0)	21 (10.9)	8 (12.3)	3 (20.0)	2 (7.7)	1 (25.0)
Cerebrovascular disease	52 (14.6)	6 (15.8)	1 (6.7)	32 (16.6)	4 (6.2)	3 (20.0)	6 (23.1)	–
Neuromuscular disease	7 (2.0)	3 (7.9)	–	2 (1.0)	1 (1.5)	–	1 (3.8)	–
Chronic lung disease	66 (18.5)	5 (13.2)	4 (26.7)	37 (19.2)	12 (18.5)	6 (40.0)	2 (7.7)	–
Chronic kidney disease	25 (7.0)	2 (5.3)	1 (6.7)	18 (9.3)	2 (3.1)	–	2 (7.7)	–
Chronic liver disease	25 (7.0)	2 (5.3)	–	7 (3.6)	13 (20.0)	1 (6.7)	2 (7.7)	–
Solid malignancy	55 (15.4)	7 (18.4)	2 (13.3)	25 (13.0)	12 (18.5)	3 (20.0)	6 (23.1)	–
Hematologic malignancy	6 (1.7)	–	1 (6.7)	3 (1.6)	1 (1.5)	–	1 (3.8)	–
BM transplantation	1 (0.3)	–	–	1 (0.5)	–	–	–	–
Organ transplantation	2 (0.6)	1 (2.6)	–	–	1 (1.5)	–	–	–
Autoimmune disease	14 (3.9)	3 (7.9)	–	9 (4.7)	2 (3.1)	–	–	–
Pregnancy	32 (9.0)	7 (18.4)	1 (6.7)	9 (4.7)	12 (18.5)	1 (6.7)	1 (3.8)	1 (25.0)
ILI symptoms								
Fever (°C)	38.3 (37.9–38.8)	38.5 (38.2–38.9)	38.3 (37.65–38.7)	38.1 (37.5–38.8)	38.1 (37.6–38.5)	38.5 (38.0–39.0)	38.75 (38.0–39.0)	38.3 (37.85–39.0)
Cough	1,724 (86.9)	245 (84.5)	63 (81.8)	643 (87.5)	246 (87.5)	37 (84.1)	71 (88.8)	419 (88.0)
Sore throat	1,067 (53.8)	204 (70.3)	50 (64.9)	335 (45.6)	185 (65.8)	32 (72.7)	46 (57.5)	215 (45.2)
Rhinorrhea/Nasal obstruction	1,092 (55.1)	215 (74.1)	41 (53.2)	308 (41.9)	179 (63.7)	26 (59.1)	60 (75.0)	263 (55.3)
Influenza vaccination (2011–2012)	430 (21.7)	74 (25.5)	23 (29.9)	145 (19.7)	71 (25.3)	8 (18.2)	24 (30.0)	85 (17.9)

DM, diabetes mellitus; BM, bone marrow; ILI, influenza-like illness.

### Distribution of Influenza and Other Respiratory Viruses in the Patients with ILI

**Figure 2:**
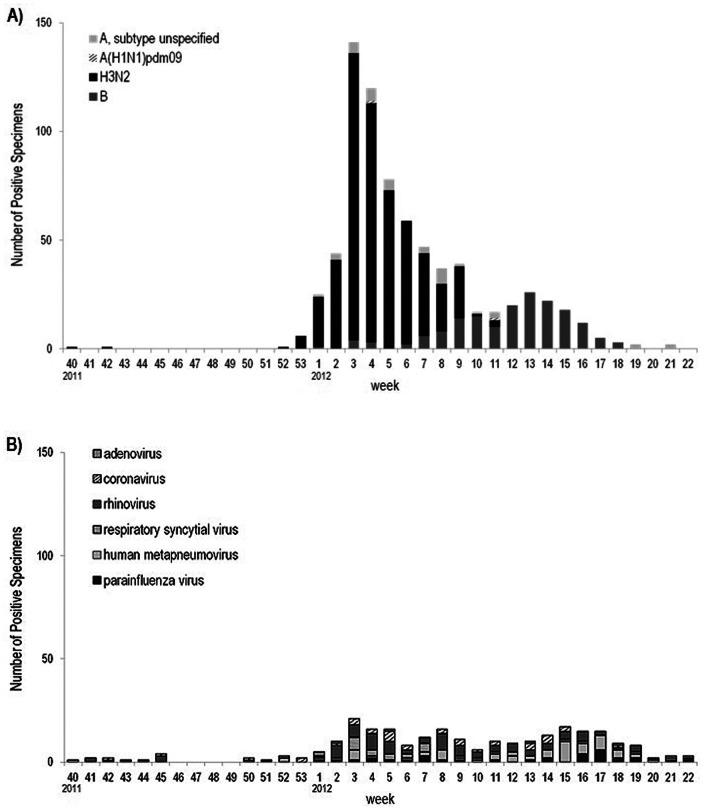
Respiratory viruses epidemics in patients with ILI during the 2011–2012 season. (A) Influenza viruses; (B) Respiratory viruses other than influenza viruses.

Median time from initial symptom onset to sample collection was 1 day (IQR 1–3). Among 1,983 respiratory specimens from ILI patients, 1,033 samples (52.1%) were positive for at least one respiratory virus, and a total of 1,100 respiratory viruses were detected (Figure 2, Table 2). Influenza A virus was the predominant agent (677, 61.5%) among the respiratory viruses detected in ILI patients. In the 2011–2012 season, H3N2 was prominent (606, 55.1%) and A(H1N1)pdm09 virus was detected in two patients. Influenza B virus was detected in 169 patients (15.4%). The distribution of influenza viruses appeared to be bimodal. The first peak appeared during week 3, starting Jan 15 and ending Jan 21, 2012. The second peak presented in week 13, starting Mar 25 and ending Mar 31, 2012. A preponderance of influenza A virus was found during the first peak, while influenza B virus was dominant during the second peak.

**Table 2:** Respiratory viruses detected in ILI patients from week 40, 2011 through week 22, 2012.

	Total (n = 1,100)	Hospital A (n = 176)	Hospital B (n = 46)	Hospital C (n = 337)	Hospital D (n = 176)	Hospital E (n = 23)	Hospital F (n = 58)	Hospital G (n = 284)
Influenza A	677 (61.5)	110 (62.5)	35 (76.1)	227 (67.4)	102 (58.0)	19 (82.6)	44 (75.9)	140 (49.3)
H3N2	606 (55.1)	102 (58.0)	33 (71.7)	214 (63.5)	85 (48.3)	19 (82.6)	44 (75.9)	109 (38.4)
A(H1N1)pdm09	2 (0.2)	1 (0.6)	–	–	1 (0.6)	–	–	–
unspecified	69 (6.3)	7 (4.0)	2 (4.3)	13 (3.9)	16 (9.1)	–	–	31 (10.9)
Influenza B	169 (15.4)	39 (22.2)	2 (4.3)	38 (11.3)	42 (23.9)	1 (4.3)	1 (1.7)	46 (16.2)
Adenovirus	7 (0.6)	1 (0.6)	2 (4.3)	–	–	–	1 (1.7)	3 (1.1)
Rhinovirus	86 (7.8)	12 (6.8)	4 (8.7)	27 (8.0)	8 (4.5)	1 (4.3)	5 (8.6)	29 (10.2)
Respiratory syncytial virus A	26 (2.4)	–	–	9 (2.7)	2 (1.1)	1 (4.3)	2 (3.4)	12 (4.2)
Respiratory syncytial virus B	4 (0.4)	–	1 (2.2)	1 (0.3)	2 (1.1)	–	–	–
Human metapneumovirus	61 (5.5)	4 (2.3)	1 (2.2)	19 (5.6)	11 (6.3)	–	1 (1.7)	25 (8.8)
Coronavirus 229E/NL63	10 (0.9)	–	–	3 (0.9)	1 (0.6)	1 (4.3)	3 (5.2)	2 (0.7)
Coronavirus OC43	26 (2.4)	6 (3.4)	–	4 (1.2)	3 (1.7)	–	–	13 (4.6)
Parainfluenza virus 1	7 (0.6)	1 (0.6)	–	1 (0.3)	3 (1.7)	–	–	2 (0.7)
Parainfluenza virus 2	9 (0.8)	1 (0.6)	1 (2.2)	3 (0.9)	–	–	1 (1.7)	3 (1.1)
Parainfluenza virus 3	18 (1.6)	2 (1.1)	–	5 (1.5)	2 (1.1)	–	–	9 (3.2)
Parainfluenza virus 4	–	–	–	–	–	–	–	–
Enterovirus	–	–	–	–	–	–	–	–
Bocavirus	–	–	–	–	–	–	–	–

Among 1,100 respiratory viruses, 254 (23.1%) were non-influenza respiratory viruses. Rhinovirus was detected in 86 patients (7.8%), and it circulated throughout the entire study period, excluding November and December. Human metapneumovirus was found in 61 (5.5%) patients. It appeared around late winter, and the epidemic peak presented during spring (week 15: Apr 8 to 14). Most RSV cases occurred in winter, but there were some episodes during spring. There was not a single detection of bocavirus or enterovirus.

### Distribution of Influenza and Other Respiratory Viruses in the Patients with ILI by Age Group

**Figure 3:**
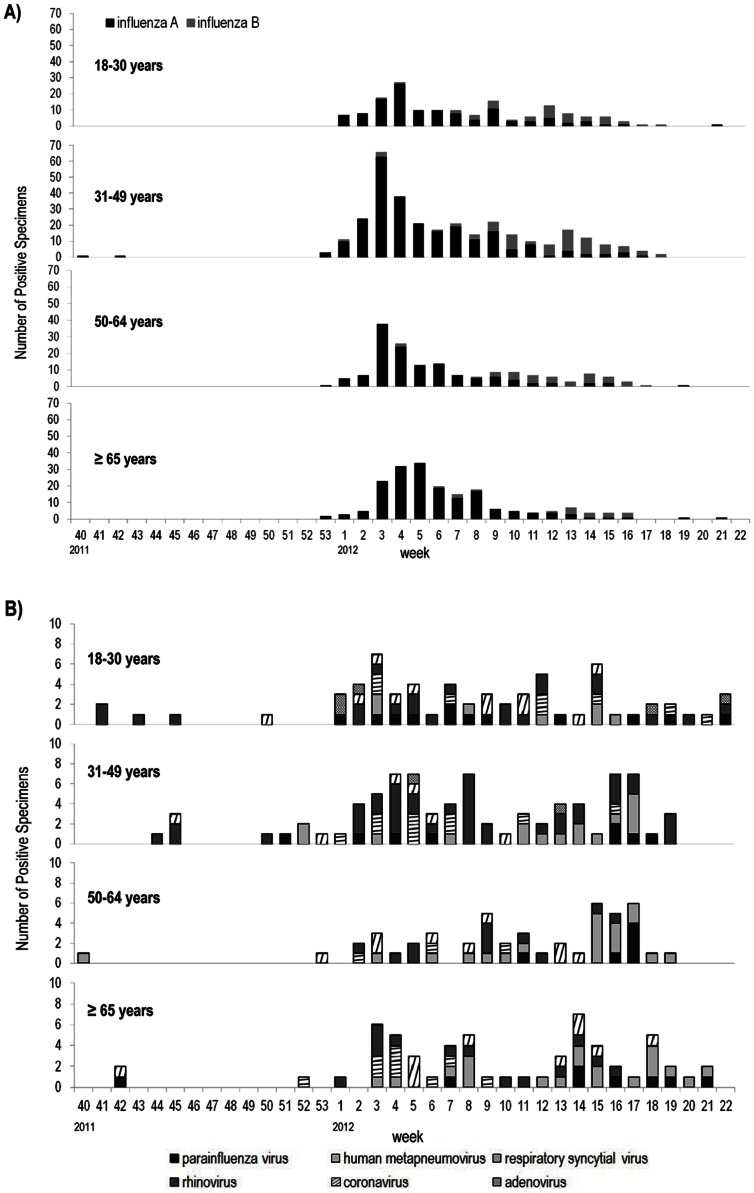
Respiratory viruses epidemics of ILI patients during the 2011–2012 season according to age group. (A) Influenza viruses; (B) Respiratory viruses other than influenza viruses.

The epidemic peak of influenza was divergent according to age group (Figure 3). It presented earlier in patients aged 31–49 years and 50–64 years during week 3 (Jan 15 to 21), during week 4 (Jan 22 to 28) in the 18–30 years group, and during week 5 (Jan 29 to Feb 4) in patients aged ≥65 years.

The proportions of other respiratory viruses were different according to age group also. The prevalence of human metapneumovirus was higher in patients more than 50 years old: 3.7% (23/630) in 18–49 years group; 8.1% (38/470) in patients aged ≥50 years. Adenovirus was detected only in patients aged less than 50 years old.

### Co-incidence Cases

There were 58 cases (5.6%) in which two or more respiratory viruses were detected from respiratory specimens. In 52 cases, two respiratory viruses were detected concurrently (Table 3). Five patients with influenza A also were found to have two other viruses: rhinovirus and RSV, rhinovirus and adenovirus, rhinovirus and parainfluenza virus, influenza B virus and rhinovirus, and influenza B virus and parainfluenza virus. In one case, six respiratory viruses were found simultaneously: influenza A virus, influenza B virus, rhinovirus, RSV A, coronavirus OC43, and human metapneumovirus. Rhinovirus (19/58, 32.8%) was the most frequently detected concurrent virus in influenza patients. There were 11 cases of co-incidence of influenza A and B.

**Table 3:** Numbers of cases in which two respiratory viruses were detected simultaneously from respiratory specimens of ILI patients.

	InfluenzaA	InfluenzaB	Rhinovirus	Respiratorysyncytial virus	Coronavirus	Humanmetapneumovirus	Adenovirus	Parainfluenzavirus
Influenza A	–	8	10	7	7	5	3	1
Influenza B	–	–	3	1	0	2	0	1
Rhinovirus	–	–	–	0	0	1	1	1
Respiratorysyncytial virus	–	–	–	–	0	0	0	0
Coronavirus	–	–	–	–	–	0	0	0
Humanmetapneumovirus	–	–	–	–	–	–	0	1
Adenovirus	–	–	–	–	–	–	–	0
Parainfluenza virus	–	–	–	–	–	–	–	–

A part of cells was expressed with a hyphen to avoid duplication of data.

In comparison of demographic characteristics between patients with single virus and those with multiple viruses in respiratory specimens, patients with hematologic malignancy (1.1% vs. 16.7%, *P* = 0.02) and organ transplantation recipients (0% vs. 8.3%, *P* = 0.04) had higher rate of co-incidence (Table 4). However, there was no significant difference in clinical outcomes including hospitalization rate and complications.

**Table 4:** Comparison of demographic findings and clinical outcome between patients with single virus and patients with multiple respiratory viruses in respiratory specimens.

	Single virus (n = 975)	Multiple virus (n = 58)	*P*
Influenza vaccination	222 (24.5)	11 (22.9)	1.00
Comorbidity	172 (17.6)	8 (13.8)	0.59
DM	67 (25.7)	1 (8.3)	0.31
Cardiovascular disease	24 (9.2)	2 (16.7)	0.32
Cerebrovascular disease	25 (9.6)	2 (16.7)	0.34
Neuromuscular disease	3 (1.1)	0 (0)	1.00
Chronic lung disease	31 (11.9)	1 (8.3)	1.00
Chronic kidney disease	12 (4.6)	0 (0)	1.00
Chronic liver disease	13 (5.0)	0 (0)	1.00
Solid malignancy	28 (10.7)	1 (8.3)	1.00
Hematologic malignancy	3 (1.1)	2 (16.7)	0.02
BM transplantation	1 (0.4)	0 (0)	1.00
Organ transplantation	0 (0)	1 (8.3)	0.04
Autoimmune disease	3 (1.1)	1 (8.3)	0.17
Pregnancy	15 (5.7)	0 (0)	1.00
Outcome			
Pneumonia	12 (1.2)	1 (1.7)	0.53
Hospitalization	57 (5.8)	2 (3.4)	0.77
ICU admission	1 (0.1)	0 (0)	1.00
Death	1 (0.1)	0 (0)	1.00

DM, diabetes mellitus; BM, bone marrow; ICU, intensive care unit.

## Discussion

This study is the first extensive laboratory surveillance of the etiology of respiratory viruses in ILI patients through teaching hospital-based influenza surveillance in South Korea. The influenza A virus was the predominant agent and the majority of influenza A virus was A(H3N2) during the 2011–2012 season. In addition, 23.1% of the respiratory viruses detected in respiratory specimens from adult patients with ILI were viruses other than influenza viruses.

The respiratory virus detection rates in respiratory specimens from ILI patients ranged from 15.6% to 78.7%, depending on the study period, the characteristics of the study population including age, type of specimens, circulating viruses, and analysis method [7]–[11]. At least one virus was detected in 52.1% of samples in this study. Influenza virus was the predominant agent, and this finding is consistent with other reports [7]–[9]. Since ILI is a clinical definition designed to detect potential influenza cases, the use of ILI as a case-definition makes influenza viruses the viruses most likely to be identified [9]. This case-definition is suitable for the purpose of HIMM surveillance, which is focused on the detection of influenza. Yu et al. investigated respiratory viral etiology in adults with acute respiratory tract infections visiting an ED in China, and the parainfluenza virus was found to be the dominant agent [12]. In their study, enrollment criteria included respiratory symptoms, a body temperature above 37.5°C, and a normal or low leukocyte count, but not radiographic abnormalities on chest [12]. There is a difference in the respiratory viral etiology between children and adults. RSV was the most prevalent virus and was associated with substantial morbidity in children with respiratory virus infection [13]–[15]. In this research, the study population included only adult patients, and the viral etiology of pediatric patients with ILI was not evaluated.

The co-incidence rates of respiratory viruses in ILI patients vary according to study (0.7–15.3%) [7]–[11]. In this study, multiple respiratory viruses were detected in 5.6% of positive specimens and 2.9% of total ILI patient samples. The clinical significance of the co-incidence of respiratory viruses has not been clearly determined. In infants with bronchiolitis, dual viral infection was a risk factor for ICU admission and co-infection with human metapneumovirus and RSV was strongly associated with disease severity [16], [17]. Additionally, viral co-infections were related to an increased probability for hospitalization in children with respiratory infection [14]. However, the impact of detection of multiple respiratory viral infections on clinical outcome has rarely been investigated in adults. In this study, the disease severity or outcome were not different significantly between patients with single virus and patients with multiple respiratory viruses. In addition, the detection of viral nucleic acid in respiratory specimens does not always suggest that it is the causative agent of the apparent infection [18]. Among respiratory viruses, rhinovirus was the most common virus detected in influenza patients. In one patient, six respiratory viruses were detected simultaneously. This 20 years old female patient was previously healthy, and the outcome was good without hospitalization or any complication or sequelae. Among 58 co-incidence cases, two patients were hospitalized (influenza A virus and rhinovirus, influenza A virus and RSV A). Further research on viral interference, especially between influenza virus and rhinovirus, is required in both animal models and humans.

This study has some limitations. First, the surveillance population was limited to adults who visited an ED with ILI. Second, the evaluation of the causative agents of ILI covered only 15 respiratory viruses, and other bacterial or viral pathogens which can cause acute febrile respiratory illnesses were not investigated. Third, there is a possibility that a low viral titer could not be detected by RT-PCR. Fourth, patients with acute febrile illness but without respiratory illness could be enrolled in this study because the recruitment depended on whether cases met the established ILI criteria or not. Finally, this study described only the result of laboratory data. However, this study is valuable for understanding the respiratory viral etiology of ILI patients during the 2011–2012 season. This study is noteworthy in that it is the first extensive laboratory surveillance of adult ILI patients through a teaching hospital-based influenza surveillance scheme in South Korea.
